# Approaches for the modulation of mechanosensitive MscL channel pores

**DOI:** 10.3389/fchem.2023.1162412

**Published:** 2023-03-15

**Authors:** Benjamin J. Lane, Christos Pliotas

**Affiliations:** ^1^ Astbury Centre for Structural Molecular Biology, School of Biomedical Sciences, Faculty of Biological Sciences, University of Leeds, Leeds, United Kingdom; ^2^ School of Biological Sciences, Faculty of Biology, Medicine and Health, Manchester Academic and Health Science Centre, The University of Manchester, Manchester, United Kingdom; ^3^ Manchester Institute of Biotechnology, The University of Manchester, Manchester, United Kingdom

**Keywords:** MscL, mechanosensitive channel, modulators, antibiotics, agonists, membrane pores

## Abstract

MscL was the first mechanosensitive ion channel identified in bacteria. The channel opens its large pore when the turgor pressure of the cytoplasm increases close to the lytic limit of the cellular membrane. Despite their ubiquity across organisms, their importance in biological processes, and the likelihood that they are one of the oldest mechanisms of sensory activation in cells, the exact molecular mechanism by which these channels sense changes in lateral tension is not fully understood. Modulation of the channel has been key to understanding important aspects of the structure and function of MscL, but a lack of molecular triggers of these channels hindered early developments in the field. Initial attempts to activate mechanosensitive channels and stabilize functionally relevant expanded or open states relied on mutations and associated post-translational modifications that were often cysteine reactive. These sulfhydryl reagents positioned at key residues have allowed the engineering of MscL channels for biotechnological purposes. Other studies have modulated MscL by altering membrane properties, such as lipid composition and physical properties. More recently, a variety of structurally distinct agonists have been shown bind to MscL directly, close to a transmembrane pocket that has been shown to have an important role in channel mechanical gating. These agonists have the potential to be developed further into antimicrobial therapies that target MscL, by considering the structural landscape and properties of these pockets.

## Introduction

In cells, molecules are exchanged between membrane compartments by transporters and channel proteins (Kung et al., 2010). Channel proteins gate in response to several activators including changes in membrane potential, or the presence of a specific ligand (Martinac et al., 2009; Alexander et al., 2011). The initial identification of an ion channel that gates in response to mechanical force provoked extensive investigation in this area (Guharay and Sachs, 1984). Mechanosensitive (MS) channels have an intrinsic ability to sense and respond to changes in bilayer tension, allowing cells to sense mechanical stimuli in their environment (Ranade et al., 2015). Bacterial MS channels sense changes in lateral tension generated in the membrane during hypoosmotic shock, acting as the safety-valves that release solutes to prevent cell lysis (Booth et al., 2007; Rasmussen, 2016). Bacterial MS channels can be divided into two major structural families: MscL and the MscS-like superfamily (MscS, MscM, MscK, YbiO, YnaI, YbdG) (Booth, 2014). MscL was the first MS channel to be identified (Lett et al., 1994) and is the last resort osmolyte-release system in bacteria and archaea. A structure of MscL from *M. Tuberculosis* (TbMscL) was the first to be solved *via* x-ray crystallography (Chang et al., 1998). TbMscL, along with *Escherichia coli* MscL (EcMscL), for which only a structure of its isolated cytoplasmic domain has been reported, are the most well studied (Walton and Rees, 2013). Despite extensive research focused on MscL, complex mechanisms related to the channel are still being uncovered. It was recently shown that MscL excretory activity is regulated by alternative ribosome-rescue factor (arfA) sRNA, linking osmotic and translational stress responses (Pratama, 2022).

The TbMscL structure showed that the protein was composed of an amphipathic helix (S1) at the N-terminus of the protein on the cytoplasmic side of the membrane, two transmembrane helices (TM1 and TM2) and a cytosolic helix at the C-terminus (Chang et al., 1998; Wang and Blount, 2023). TM1 and TM2 are connected by a large periplasmic loop, and there are connecting loops between the cytoplasmic helical bundle and the bottom of TM2. The pore is lined by TM1 from each subunit (Chang et al., 1998; Walton et al., 2015). The TM1 helix from each monomer contacts two other TM1 helices from adjacent monomers, and two TM2 helices, one from the same monomer and one from a neighbouring subunit. TM1 helices are tilted from the plane of the bilayer and this gives MscL a pore that opens like a camera iris (Betanzos et al., 2002). This non-selective pore opens to an estimate diameter of ∼30 Å, and results in a conductance of ∼3 nS (Lett et al., 1994; Cruickshank et al., 1997; Wang and Blount, 2023). The open structure of MscL has remained elusive, however a structure of MscL in an expanded state from archaea has been reported (Li et al., 2015). The channels only known natural stimuli is lateral tension in the bilayer, which is not trivial in detergent aqueous buffer and a natural physiological agonist has not been identified. As a result, researchers have employed a variety of approaches to modulate the channel to stabilize an activated state through the use of molecular means and/or modifications, providing insights into the gating mechanism of MscL, and highlighting the protein as a target for the development of antimicrobials, given its absence from eukaryotes.

## Modulation of MscL through mutations and post-translational modifications

**FIGURE 1:**
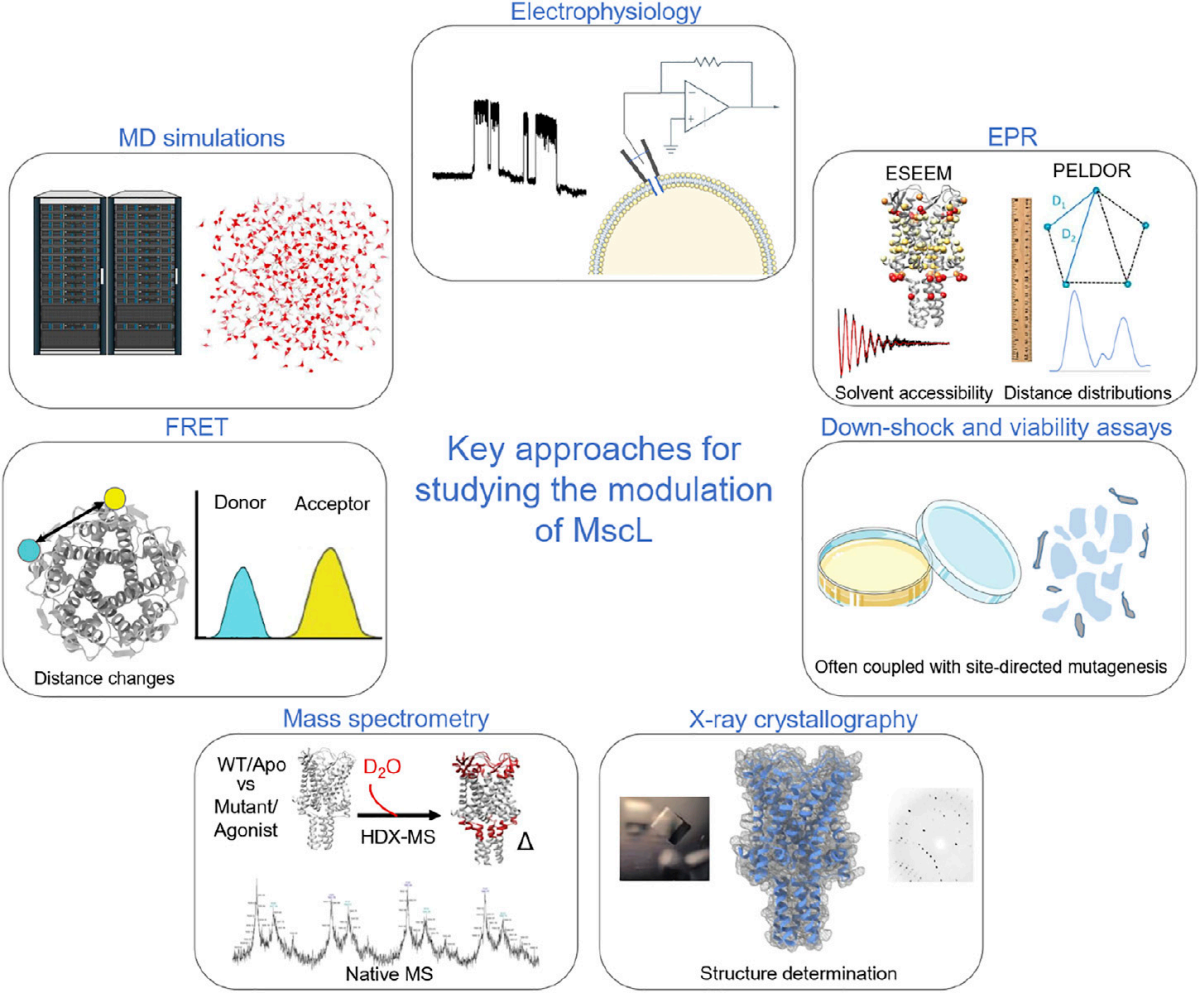
Key methods used to probe and investigate the modulation of MscL. Electrophysiology is a key functional methodology for understanding how modulation in the form of mutations, post-translational modification, agonists and indirect modulators alter the functional parameters of the protein. In early studies of MscL, this was often paired with cell viability and osmotic down-shock assays. X-ray crystallography allowed the visualisation of the structure of TbMscL. Pulsed EPR techniques, such as PELDOR and ESEEM, allow the structural dynamics of the protein to be followed through Å resolution distance measurements and by monitoring changes in solvent accessibility (Kapsalis et al., 2019; Wang et al., 2022). HDX-MS also informs on changes in solvent accessibility, albeit at lower resolution than ESSEEM spectroscopy (Lane et al., 2022; Wang et al., 2022). Native mass spectrometry was key to determining the effect of detergents and lipids on channel stoichiometry (Reading et al., 2015), while ion mobility mass spectrometry defined key subconducting states of MscL in response to cysteine-specific post-translation modification in the pore (Konijnenberg et al., 2020). MD simulations have been crucial in understanding modulation and mechanism of MscL (Bavi et al., 2016; Wray et al., 2016; Melo et al., 2017; Wang et al., 2022). Finally, fluorescence resonance energy transfer (FRET) was used in establishing a helix-tilt model for MscL following opening of the channel using LPC (Wang et al., 2014).

Early methods for the modulation of MscL relied on mutational studies that led to gain-of-function (GOF) or loss-of-function (LOF) characteristics, many of which gave insights into the gating mechanism of the channel. An early study used random mutagenesis as part of a forward genetics approach (Ou et al., 1998). This identified mutations that led to increased activation of MscL and subsequent screening of gain-of-function mutants showed these were mostly hydrophilic and present in the first TM helix (TM1) (Ou et al., 1998). Later, the structure of the TbMscL was reported which demonstrated that many of the mutations were present around the pore constriction site (Chang et al., 1998; Wang and Blount, 2023). Random mutagenesis studies have been key in identifying mutations that increase the activity of channels (GOF) or increase the barriers or completely abolish gating. A high-throughput screen of 348 mutations allowed the identification of 5 new GOF mutations and 45 new LOF mutations (Maurer and Dougherty, 2003). Analysis of the mutations highlighted TM2 as being functionally significant (Maurer and Dougherty, 2003). A later study looked at the function of LOF mutants generated by a random approach (Yoshimura et al., 2004). Patch-clamp measurements and hypoosmotic shock experiments (Figure 1) showed that the replacement of hydrophobic residues at the end of TM1 and TM2 with hydrophilic residues would eradicate the ability of MscL to open in response to membrane tension.

In the same study, systematic asparagine scanning mutagenesis also identified residues at lipid-protein interface on the periplasmic side that were essential for MscL gating (Yoshimura et al., 2004). Systematic scanning or selective mutagenesis studies can also identify mutation that modulate MscL channel behaviour. G22 is a residue within the constriction site of MscL, that was identified in a random mutagenesis screen as a severe GOF mutation (Ou et al., 1998). Subsequent systematic mutation of G22 to the other 19 common mutations was done to analyse the effect on channel gating and cell growth. Hydrophilic substitutions increased while hydrophobic substitution decreased the pressure threshold (Yoshimura et al., 1999). This suggested that G22 must move from a hydrophobic environment, through a hydrophilic environment, upon gating. A G22N mutant of MscL displayed spontaneous opening in liposomes and was consistent with the stabilization of an expanded state (Yoshimura et al., 2008). Additional hydrophilic mutations that lead to GOF behaviour within TM1 were identified using cysteine scanning (Levin and Blount, 2004). These mutations lower the energy barrier for gating, consistent with TM1 separation being coupled with channel conductance (Levin and Blount, 2004). GOF mutations in TM2 did not correlate with hydrophilicity, and it was thought that these residues may be important in maintaining key lipid interactions, which was confirmed later by high resolution spectroscopic studies (Kapsalis et al., 2019). Another approach used histidine substitution of residues that are predicted to line the pore in different conformational states, and looked at the ability of Ni^2+^, Cd^2+^ or Zn^2+^ ions to alter channel gating thresholds (Iscla et al., 2004).

Iscla et al. (2008) used a scanning approach where cysteine mutations were paired with sulfhydryl reagents that conferred different charges and/or hydrophobicity to different sites, allowing them to identify key residues that modulated channel function and had roles in channel gating (Iscla et al., 2015a). Cysteine scanning of the S1 domain of EcMscL showed the region was important for normal channel function, even though none of the cysteine mutations led to a non-functional MscL channel *in vivo* (Iscla et al., 2008). Further work on the S1 domain using continuous wave electron paramagnetic resonance (cwEPR) spectroscopy and molecular dynamics (MD) simulations showed that lipids strongly interact with the N-terminus during channel expansion, leading to the proposal of the ‘dragging’ model (Bavi et al., 2016; Bavi et al., 2017). The S1 amphipathic helix is directly connected to the pore-lining segment in MscL, but this is not the case for other mechanosensitive ion channels (Kefauver et al., 2020). Coupling of a cysteine mutation at a pore-lining residue with chemical modification to introduce a molecule has also allowed the engineering of MscL so that the channel responds to a variety of stimuli such as pH and light, with potential applications in biotechnology (Koçer et al., 2005; Koçer et al., 2006; Yang et al., 2018; Yang et al., 2021). A pH sensitive channel was generated through the attachment of sulfhydryl-reactive modulators to a G22C mutant in the pore of EcMscL (Koçer et al., 2006; Yang et al., 2018; Yang et al., 2021). Many of the mutations that led to increases in the activation of MscL discussed here relied on the modification of pore-lining residues (Yoshimura et al., 1999; Iscla et al., 2004). However, as these residues were inaccessible to lipid molecules, they failed to report on an allosteric lipid-mediated activation of MscL which occurs in its natural environment. Additionally, many early studies mostly depended on electrophysiology and cell viability/growth assays (Figure 1), and therefore they lacked the ability to directly detect gating and dynamics of MscL at a molecular level.

The introduction of an L89W mutation on the TM2 helix in TbMscL offered insights into the gating mechanism of bacterial mechanosensitive channels in response to tension in the membrane. Previous studies of MscS have led to the development of the lipid-moves-first model where the number lipid acyl chains occupying TM pockets determined the conformational state of the protein (Pliotas et al., 2012; Pliotas et al., 2015; Pliotas and Naismith, 2017). Increases in lateral tension were thought to cause the movement of lipids from the pockets to the bulk bilayer, destabilizing the closed structure. This model was extended to MscL as it also has TM pockets and pulsed electron paramagnetic resonance (EPR) spectroscopic studies suggested a similar mechanical sensing mechanism as MscS (Kapsalis et al., 2019; Kapsalis et al., 2020). The introduction of the mutation L89W at the entrance to these TM pockets stabilized an expanded and subconducting state of TbMscL (Kapsalis et al., 2019). In EcMscL, L89W corresponds to M94 when aligning the sequence using CLUSTALX (Chang et al., 1998), and to A95 when using Protein BLAST. Pulsed electron-electron double resonance (PELDOR, also known as DEER) (Pliotas, 2017; Bordignon et al., 2019; Hartley et al., 2020; Ackermann et al., 2017) and electron spin echo envelope modulation (ESEEM) spectroscopy (Hartley et al., 2020; Lane et al., 2022) are powerful tools in the assessment of conformation in integral membrane proteins (Figure 1). PELDOR measurements showed a conformational change consistent with an expanded state in TbMscL (Kapsalis et al., 2019). This approach also allowed structural alignment of TbMscL and EcMscL in lipid nanodiscs, showing that L89W (TbMscL) structurally corresponds to M94 in *E. coli* (Kapsalis et al., 2020). PELDOR spectroscopy allows for high-resolution distance measurements which can be used to follow folding and conformational changes, and was utilized to assess the correct folding of MscL when it was expressed in new strains designed for efficient membrane protein expression (Michou et al., 2019). The presence of the mutation also reduced the threshold required for channel conductance in electrophysiology measurements, consistent with a subconducting state. The data suggests that the presence of a bulky tryptophan or sulfhydryl modification at the entrance of lipid-accessible TM pockets caused destabilization of the closed state by hindering the penetration of lipid acyl chains into these TM pockets and highlighting the importance of this region of the channel (Kapsalis et al., 2019). The MTSSL spin label on a introduced cysteine residue, modulated channel function as seen previously for other sulfhydryl modification, but also allowed high-resolution measurements to follow conformational changes in the channel (Iscla et al., 2015b; Kapsalis et al., 2019). The expanded state was characterized further using hydrogen-deuterium exchange mass spectrometry (HDX-MS) experiments and ESEEM spectroscopy measurements, highlighting structural transitions that occur from modulation by the L89W mutation. MD simulations of TbMscL in a lipid bilayer under tension were completed to stabilize an expanded state of TbMscL in response to mechanical stimuli, which showed pore hydration (Wang et al., 2022). Comparison of the two states showed they were structurally analogous and that the mutant stabilized state was biologically relevant, further supporting the lipid-moves-first model and the importance of the region of the TM pockets in channel gating (Wang et al., 2022).

## Modulation of MscL *via* direct binding of molecules and antimicrobials

**FIGURE 2:**
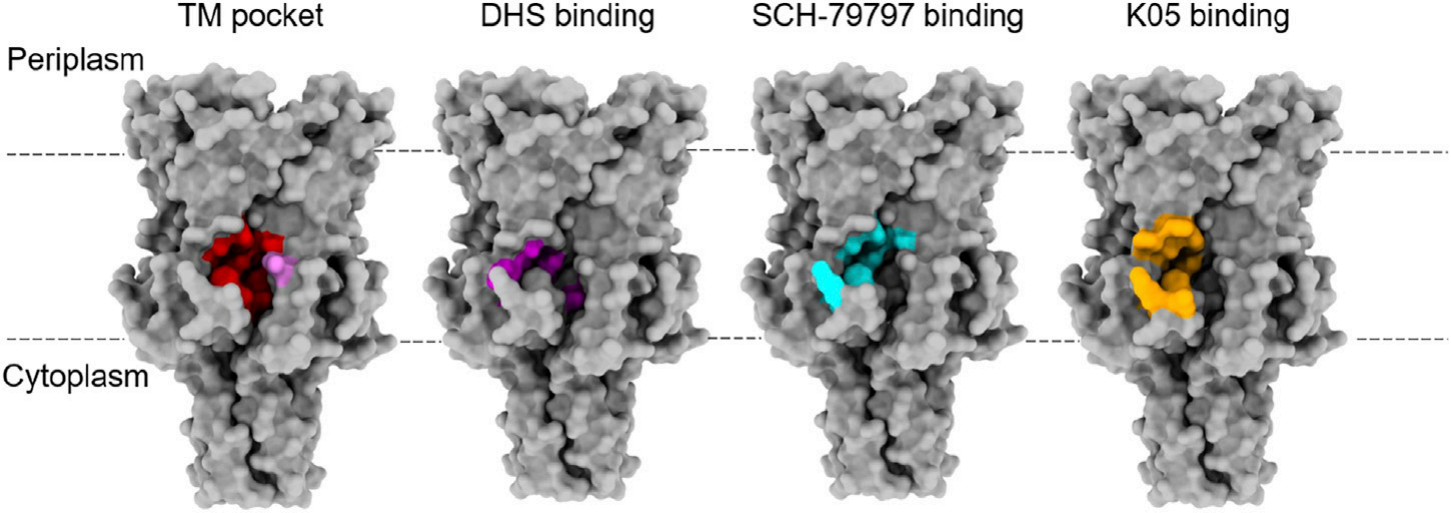
Binding sites for lipids and agonists of MscL. The TM pocket (red) of MscL is occupied by several lipid acyl chains that are proposed to determine the conformational state of the MS channel proteins, according to the lipid moves first model initially proposed for MscS and then extended to MscL (Pliotas et al., 2015; Pliotas and Naismith, 2017; Kapsalis et al., 2019). This was defined when either a bulky L89W (pink) mutation or a L89C sulfhydryl modification (MTSSL) succeeded in stabilising an expanded subconducting state of the TbMscL, consistent with the modifications at the entrance of these pockets restricting lipid acyl chain access (Kapsalis et al., 2019). This is proposed to have disrupted the link between the membrane and the channel, destabilising the closed state. Several agonists of MscL have been identified and they all bind at the interface between the S1 and TM1 region of one subunit with the TM2 of another at the membrane-cytoplasmic interface (Wray et al., 2016; Wray et al., 2020; Wray et al., 2021). These agonists all bind close to the TM pocket and so these molecules could be disrupting protein-lipid interactions that are key to determining the conformational state of the protein. Agonist studies were done on EcMscL but equivalent residues were highlighted on the structure of TbMscL (2OAR) in the absence of a structure for EcMscL.

Some chemical compounds also have been shown to modulate MS channels through direct interactions with the protein, primarily targeting the MscL protein at the cytoplasmic-membrane interface close to the region of the TM pockets (Figure 2) (Wang and Blount, 2023). The well-known antibiotic dihydrostreptomycin (DHS) crosses the membrane primarily through MscL (Wray et al., 2016). DHS also directly binds to MscL at the subunit interface near the constriction site, which causes efflux of potassium and glutamate through the open MscL, followed by the passing of DHS into the cytoplasm (Dubin et al., 1963; Wray et al., 2016). This binding site is in the same region of the TM pockets that were previously shown to have an important role in mechanosensation. MD simulations suggest that MscL does not fully open upon DHS binding but several structural changes associated with a transition towards an open channel are observed, such as a rotation of TM1 and the separation of the helices around the pore (Wray et al., 2016). Ramizol was a compound identified through an *in silico* screening approach as a MscL interactor and was shown to inhibit the growth of MscL-expressing *Staphylococcus aureus* (Iscla et al., 2015a), and in patch-clamp electrophysiology it reduced the gating threshold of MscL (Iscla et al., 2015b). However, data suggests that ramizol likely has other targets in the cell or may have some amphipathic affect (Sidarta et al., 2022). However, it has the potential to be developed as a therapeutic against bacteria and has been through pre-clinical studies (Rao et al., 2016; Sidarta et al., 2022).

Curcumin, a flavonoid polyphenol from turmeric, and SCH-79797 activate MscL and lead to membrane permeabilization (Ingolfsson et al., 2007; Tyagi et al., 2015; Teow et al., 2016; Gupta et al., 2018; Martin et al., 2020; Wray et al., 2021). SCH-79797 and its derivatives have been shown to be affective against antibiotic-resistant strains and infection models (Gupta et al., 2018). SCH-79797 and related compounds bind directly to MscL, activating the channel, and causing membrane permeabilization (Wray et al., 2021). The binding site for SCH-79797 sits at the interface of the N-terminal residues (S1) and the cytoplasmic side of TM2, which is a similar region as seen for DHS but more peripheral to the pore (Wray et al., 2021). Curcumin limits bacterial growth in a MscL-dependent way and it also increases MscL channel activity. However, a direct binding site for curcumin has not been identified meaning it may act *via* altering the biophysical properties of the membrane, e.g., by thinning of the membrane (Ingolfsson et al., 2007). The organic molecules 011A and K05 have been identified as agonists of MscL (Wray et al., 2019a; Wray et al., 2019b; Wray et al., 2020). They bind to MscL and increase its sensitivity to lateral tension (Wray et al., 2019a; Wray et al., 2020). The binding site is positioned at cytoplasmic-membrane interface with residue 97 demonstrated as being an essential residue for the binding of the compounds in EcMscL. This residue region was revealed by high-resolution PELDOR/DEER distance measurement in lipid bilayers to be structurally different in distinct MscL orthologues (i.e., EcMscL and TbMscL) suggesting subtle differences within these regions account for functional diversity between different MscL orthologues (Kapsalis et al., 2020). Additionally, these molecules increase the potency of the commonly used antibiotics dihydrostreptomycin, kanamycin, tetracycline, and ampicillin making them potentially useful as antibiotic adjuvants (Wray et al., 2019b; Wray et al., 2020; Sidarta et al., 2022). Finally, a structurally distinct small molecule, known as compound 262, was shown by *in silico* docking experiments to be a potential MscL agonist that binds to a similar pocket as 011A and K05 (Wray et al., 2022). Therefore, all currently known agonists of MscL bind close to the central pore, in or around the previously defined TM pocket which responds to the availability of lipid acyl chains, despite being structurally diverse (Figure 2) (Wang and Blount, 2023). Direct modulators of MS channels are needed in order to help with mechanistic studies of these proteins, and for the development of novel antimicrobials. However, all these studies highlight MscL as a druggable target, and there is potential for the development of new compounds that target this pocket.

## Modulation of MscL *via* membrane properties and components

Modulators can act indirectly on MS channels, such as through the modification of the physical or chemical properties of the membrane. A decrease in membrane thickness was shown to lower the activation threshold of EcMscL, while decreased membrane fluidity hampered EcMscS gating (Perozo et al., 2002; Nomura et al., 2012; Xue et al., 2020). An atomistic MD study showed that the thickness-dependent gating of MscL is likely to be driven by the hydrophobic matching of the protein to the thickness of the bilayer, largely through the interaction of F78 (TbMscL) with the membrane surface on TM2 (Katsuta et al., 2019). Coarse-grained molecular dynamic simulations of curved bilayers that were generated through the asymmetric incorporation of lysophosphatidyl choline (LPC) showed that upon asymmetric incorporation, compression occurs in upper leaflet and dilation occurs in the lower leaflet (Yoo and Cui, 2009). This is consistent with experimental studies showing asymmetric LPC incorporation can activate MscL, while the addition of cholesterol at varying concentrations to azolectin liposomes increased the membrane tension required to activate MscL (Perozo et al., 2002; Nomura et al., 2012). The addition of cardiolipin to DOPE/DOPC membranes increases the opening and closing thresholds for MscL. However, this is complicated further as in azolectin liposomes, MscL remains largely unaffected by the presence of cardiolipin. Poly-unsaturated fatty acids also lower the tension threshold of MscL (Ridone et al., 2018). MscL has also been activated in bilayer containing a photoswitchable lipid molecule (AzoPC). Light can switch AzoPC from its trans to cis state using blue light which increases lateral tension in the membrane and stabilises a subconducting state (Crea et al., 2022).

Several studies have highlighted the use of *β*-cyclodextrin (β-CD) in the modulation of MS channels. Cyclodextrins work by mimicking tension through the removal of lipids from liposomes or nanodiscs which lowers lipid density (Zhang et al., 2021). *β*-CD was used to stabilize a desensitised state of MscS from cryo-electron microscopy (cryo-EM) studies, and another study showed that cyclodextrin-induced lipid removal was also able to activate MscL despite the high tensions required for gating of this channel (Cox et al., 2021). Other amphipathic molecules, such as parabens, trinitrophenol, trifluroethanol, and fluorouracil, have indirect effects on MS channels through their intercalation into the membrane and their activation effectiveness is proportional to their hydrophobicity, but is also affected by their size and shape (Martinac et al., 1990; Nguyen et al., 2005; Kamaraju and Sukharev, 2008; Bavi et al., 2022). MD simulations of MscL in different bilayer environments in the presence of alcohols, supported by experimental efflux assays, showed that straight-chain alcohols increased channel gating periods (Melo et al., 2017). Asymmetric effects of amphipathic molecules seems to represent a general mechanism of regulation for mechanosensitive channel (Bavi et al., 2022). For MscS it was shown that parabens affect the sensitivity of MscS differently depending on whether they are applied to the cytoplasmic or periplasmic side (Kamaraju and Sukharev, 2008). On the periplasmic side, they increased sensitivity, while on the cytoplasmic side they decreased sensitivity. Alpha helical peptides, known as piscidins, have been shown to lower the activating tension of MscL and MscS in spheroplasts (Comert et al., 2019; Cetuk et al., 2020). It is thought that they act through the modification of protein-lipid boundary by inducing tension or membrane curvature. However, these peptides likely have other targets as *Escherichia coli* (*E. coli*) strains lacking MscL, MscS and MscK did not differ much from wild type *E. coli* strains in response to piscidins. Gadolinium chloride (GdCl_3_) exclusively acts as an inhibitor that blocks MS channel gating (Berrier et al., 1992; Ermakov et al., 2010). Gd^3+^ interacts with the lipid bilayer and causes it to compact, holding the MS channels in their closed state. However, it was shown that GdCl_3_ could only inhibit the gating of MscL when anionic phospholipids were present in the membrane, indicating that they may serve as a sort of receptor to facilitate the interaction of Gd^3+^ with the bilayer (Ermakov et al., 2010). Finally, the globular amphipathic peptide, GsMTx4, from spider venom is another indirect modulator of the activity of MS channels (Jung et al., 2006; Hurst et al., 2009; Kamaraju et al., 2010). When applied to the cytoplasmic side it increased channel opening in MscL and MscS (Kamaraju et al., 2010). However, when applied to the periplasmic side of a membrane patch, lower peptide concentrations of 2–4 µM decreased channel sensitivity to pressure, while at higher concentrations (>12 µM) the opposite was true (Hurst et al., 2009). However, all modulators discussed here are non-specific to the MscL protein, acting indirectly.

## Concluding remarks

Modulation of MscL through the introduction of mutations, posttranslational modifications, alteration of membrane components, and the direct modulation of binding molecules has provided insights into the mechanism of the channel protein with the largest known gated pore to date. Modulators of MS channels could be used as tools in future mechanistic studies, for the stabilization of functional states of these proteins, and for the development of biotechnological applications. Aside from this, MscL is a particularly attractive target for drug development. The channel has strong conservation among bacteria, while being structurally distinct from eukaryotic channels, and is both a direct target, but also has the potential to improve other antimicrobial therapies through cell permeabilization (Doerner et al., 2012; Sidarta et al., 2022; Wang and Blount, 2023). Overall, there is great potential for new pioneering discoveries through the modulation of bacterial MS channels in order to develop understanding of their structures, mechanisms, and functions, but also for their use within biotechnology and as targets for antimicrobial therapies.
